# Involvement of radiologists in oncologic multidisciplinary team meetings: an international survey by the European Society of Oncologic Imaging

**DOI:** 10.1007/s00330-020-07178-w

**Published:** 2020-08-24

**Authors:** Emanuele Neri, Michela Gabelloni, Tobias Bäuerle, Regina Beets-Tan, Damiano Caruso, Melvin D’Anastasi, Julien Dinkel, Laure S Fournier, Sofia Gourtsoyianni, Ralf-Thorsten Hoffmann, Marius Erik Mayerhöfer, Daniele Regge, Heinz Peter Schlemmer, Andrea Laghi

**Affiliations:** 1 Diagnostic and Interventional Radiology, Department of Translational Research, University of Pisa, Via Roma 67, 56126 Pisa, Italy; 2 Institute of Radiology, University Hospital Erlangen, Maximiliansplatz 3, 91054 Erlangen, Germany; 3 Department of Radiology, The Netherlands Cancer Institute, Amsterdam, The Netherlands; 4 Department of Radiological Sciences, Oncological and Pathological Sciences, University of Rome “Sapienza”, Sant’Andrea University Hospital, Rome, Italy; 5 Medical Imaging Department, Mater Dei Hospital, University of Malta, Valletta, Malta; 6 Department of Radiology, University Hospital, LMU Munich, Munich, Germany; 7 Radiology Department, Hôpital européen Georges Pompidou, AP-HP, Université de Paris, 20 Rue Leblanc, F-75015 Paris, France; 8 1st Department of Radiology, School of Medicine, Areteion Hospital, National and Kapodistrian University of Athens, Athens, Greece; 9 Diagnostische und Interventionelle Radiologie Universitätsklinikum Dresden, Fetscherstr. 74, 01307 Dresden, Germany; 10 Division of General and Pediatric Radiology, Department of Biomedical Imaging and Image-Guided Therapy, Medical University of Vienna, A-1090 Vienna, Austria; 11 Radiology Unit, Candiolo Cancer Institute, FPO-IRCCS, University of Torino, Strada Provinciale 142 km 3.95, 10060 Candiolo, TO Italy; 12 German Cancer Research Center (DKFZ) Foundation Under Public Law, Im Neuenheimer Feld 280, 69120 Heidelberg, Germany

**Keywords:** Interdisciplinary communication, Medical oncology, Radiologists, Surveys and questionnaires

## Abstract

**Objectives:**

Multidisciplinary tumour boards (MTBs) play an increasingly important role in managing cancer patients from diagnosis to treatment. However, many problems arise around the organisation of MTBs, both in terms of organisation-administration and time management. In this context, the European Society of Oncologic Imaging (ESOI) conducted a survey among its members, aimed at assessing the quality and amount of involvement of radiologists in MTBs, their role in it and related issues.

**Methods:**

All members were invited to fill in a questionnaire consisting of 15 questions with both open and multiple-choice answers. Simple descriptive analyses and graphs were performed.

**Results:**

A total of 292 ESOI members in full standing for the year 2018 joined the survey. Most respondents (89%) declared to attend MT-Bs, but only 114 respondents (43.9%) review over 70% of exams prior to MTB meetings, mainly due to lack of time due to a busy schedule for imaging and reporting (46.6%). Perceived benefits (i.e. surgical and histological feedback (86.9%), improved knowledge of cancer treatment (82.7%) and better interaction between radiologists and referring clinicians for discussing rare cases (56.9%)) and issues (i.e. attending MTB meetings during regular working hours (71.9%) and lack of accreditation with continuing medical education (CME) (85%)) are reported.

**Conclusions:**

Despite the value and benefits of radiologists’ participation in MTBs, issues like improper preparation due to a busy schedule and no counterpart in CME accreditation require efforts to improve the role of radiologists for a better patient care.

**Key Points:**

*• Most radiologists attend multidisciplinary tumour boards, but less than half of them review images in advance, mostly due to time constraints.*

*• Feedback about radiological diagnoses, improved knowledge of cancer treatment and interaction with referring clinicians are perceived as major benefits.*

*• Concerns were expressed about scheduling multidisciplinary tumour boards during regular working hours and lack of accreditation with continuing medical education.*

## Introduction

A multidisciplinary team is defined by the National Cancer Institute as a “treatment planning approach in which a number of doctors who are experts in different specialties (disciplines) review and discuss the medical condition and treatment options of a patient” [1]. Oncologic multidisciplinary teams are also known as “multidisciplinary tumour boards” (MTBs) and their core composition may vary depending on the cancer type, but it generally includes clinical oncologists, surgeons, pathologists, diagnostic and interventional radiologists, palliative care physicians and radiation oncologists [2, 3].

MTBs are required to manage a patient from diagnosis to treatment (potentially leading to better outcomes) and to discuss patients’ eligibility for clinical trials [4, 5]. Furthermore, MTBs improve communication between different specialties and are a good opportunity for trainees to learn, and for members to update their professional knowledge. While the function of MTBs is not primarily educational, they also help to deepen the level of knowledge of participants over time [6, 7].

However, many problems arise in practice regarding logistic issues, administrative support, lack of documentation and time management [8, 9]. Time commitment depends on the frequency of meetings, their duration, the number and the complexity of the cases examined, and the time and effort for image reviewing. Moreover, specialists like radiologists or pathologists involved in the management of different types of cancer usually spend a substantial amount of time to prepare meetings, which adds significantly to their workload [10].

In 2014, the Royal College of Radiologists (RCR) published a document which highlights the importance of radiologists in MTBs and outlines the necessary requirements for consultant radiologists and radiology departments to participate in the meetings [11].

In view of the key role of the radiologist in MTBs and of the commitment required to participate in them, the European Society of Oncologic Imaging (ESOI) conducted a survey among its members aimed at assessing the quality and amount of radiologists’ involvement in MTBs, their role and related issues in clinical practice.

## Material and methods

The online survey was prepared by a panel of ESOI experts recruited among the members of the Board. The questionnaire was drafted by a facilitator (E.N.) and shared among the panellists in two rounds, the first aimed to get feedback on the questions proposed, and the second to reach a consensus on the final draft.

The questionnaire consisted of 15 questions (Table 1). Questions allowed a mix of free text and multiple choice answers, including contact details and affiliation of each respondent.

**Table 1:** ESOI questionnaire about the involvement of radiologists in oncologic multidisciplinary team meetings

Question #	Question text	Answer
1	What is your role in the imaging department?	
2	Do radiologists in your department attend oncologic multidisciplinary teams?	a) yes b) no
2a	Why do they not attend?	a) they are not formally invited b) they have a busy schedule for imaging and reporting c) they are not interested
3	How many radiologists usually attend MTB meetings?	
4	How do radiologists prepare for MTB meetings?	a) they receive the list of patients including imaging studies b) they receive only the list of patients without imaging studies c) they do not receive the list of patients
4a	In how many cases do radiologists review the imaging studies prior to MTB meetings?	a) 0% b) 10–30% c) 30–50% d) 50–70% e) > 70% f) all g) don’t know
4b	Which are the main obstacles for the review of imaging studies prior to MTB meetings?	a) lack of imaging studies of outpatients b) poor quality of imaging studies of outpatients c) lack of time due to a busy schedule for imaging and reporting
4c	Which are the available facilities to review the imaging studies during MTB meetings?	a) PACS workstations with monitor b) PACS workstations connected to a video projector c) portable personal computer
5	Are the radiologists included in the final multidisciplinary team report?	a) yes b) no
6	When the radiologist’s opinion differs from the primary imaging report, is a supplementary report provided during MTB meetings?	a) yes b) no
7	Is the radiologists’ attendance accounted in their regular working hours?	a) yes b) no
8	Is the radiologist’s attendance addressed in the appraisal process of the department?	a) yes b) no
9	Are MTB meetings accredited with CME?	a) yes b) no
10	Can you estimate in how many cases the attendance of radiologists changes the diagnostic strategy or refines the therapeutic decisions during MTB meetings?	a) < 25% b) 25–50% c) 50–100% d) don’t know
11	In your opinion, what are the most important benefits of MTB meetings for radiologists? (multiple choice)	a) translational research b) information about ongoing clinical trials c) improved knowledge of cancer treatment d) better interaction between radiologists and referring clinicians e) surgical and histological feedback
12	In your opinion, what are the most important deficiencies of MTB meetings for radiologists? (multiple choice)	a) lack of clarity with respect to clinical query b) absence of referring physicians c) inadequate IT resources d) insufficient documentation available e) timing of MTB meetings f) lack of time
13	Is the patient present at MTB meetings?	a) yes b) no
14	At what time of the day are MTB meetings usually held in your institution?	a) morning b) early afternoon c) late afternoon d) lunch time e) different time points
15	In your opinion, are MTB meetings useful?	a) mandatory b) very useful c) not useful

All members were invited to fill in the questionnaire with an email invitation sent by the ESOI office. A reminder was sent 2 weeks after the first invitation in order to collect the maximum number of responses.

The Google Forms® platform was used to facilitate the filling in of the questionnaire, which was available to participants via a personal web link. Answers from each respondent were exported in Microsoft Excel® format for ease of data collection and statistical analysis. Simple descriptive analyses and graphs were performed using Microsoft Excel 2018® (Microsoft Office, 2018).

## Results

**Fig. 1:**
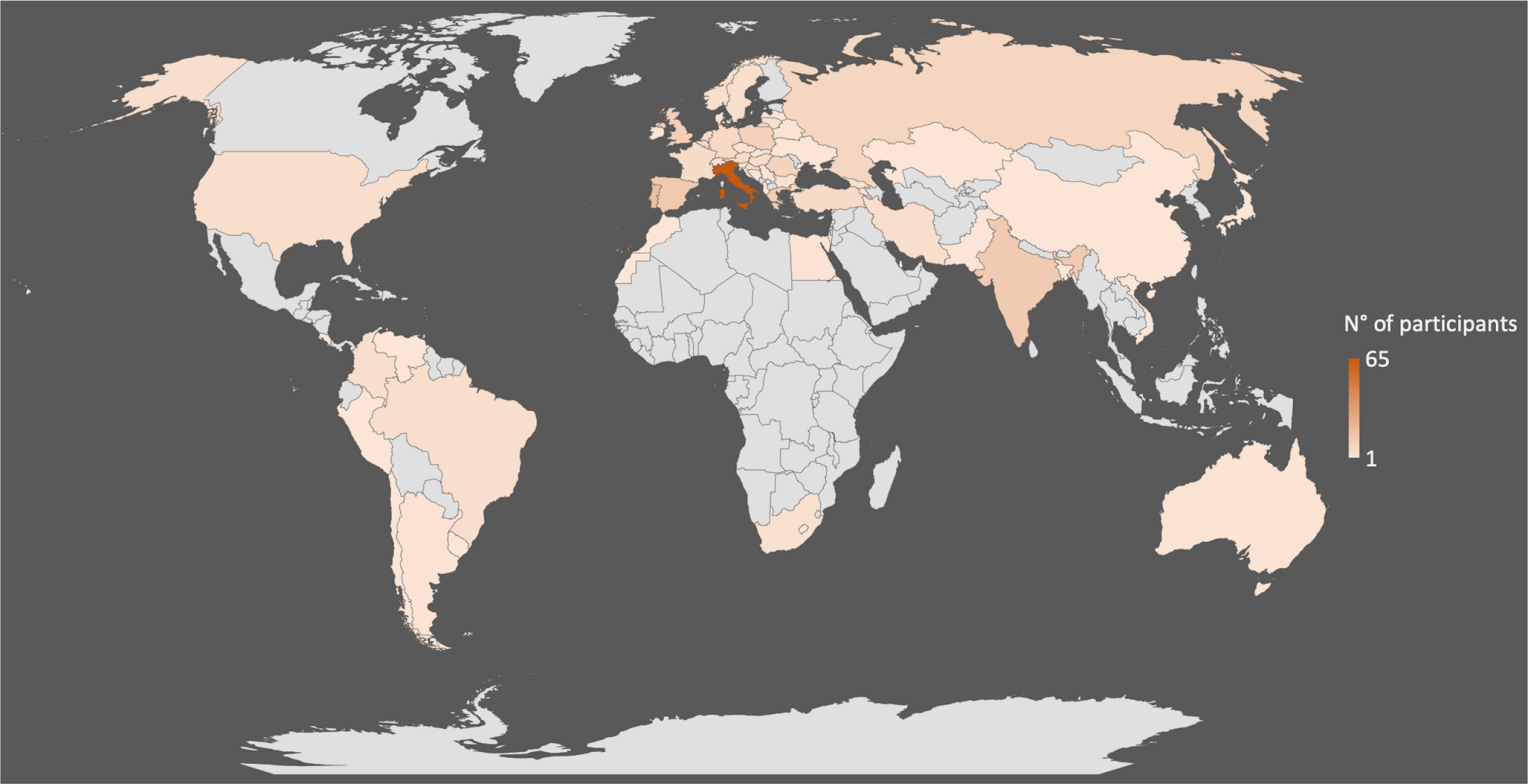
World map showing the geographic distribution of survey responders

**Fig. 2:**
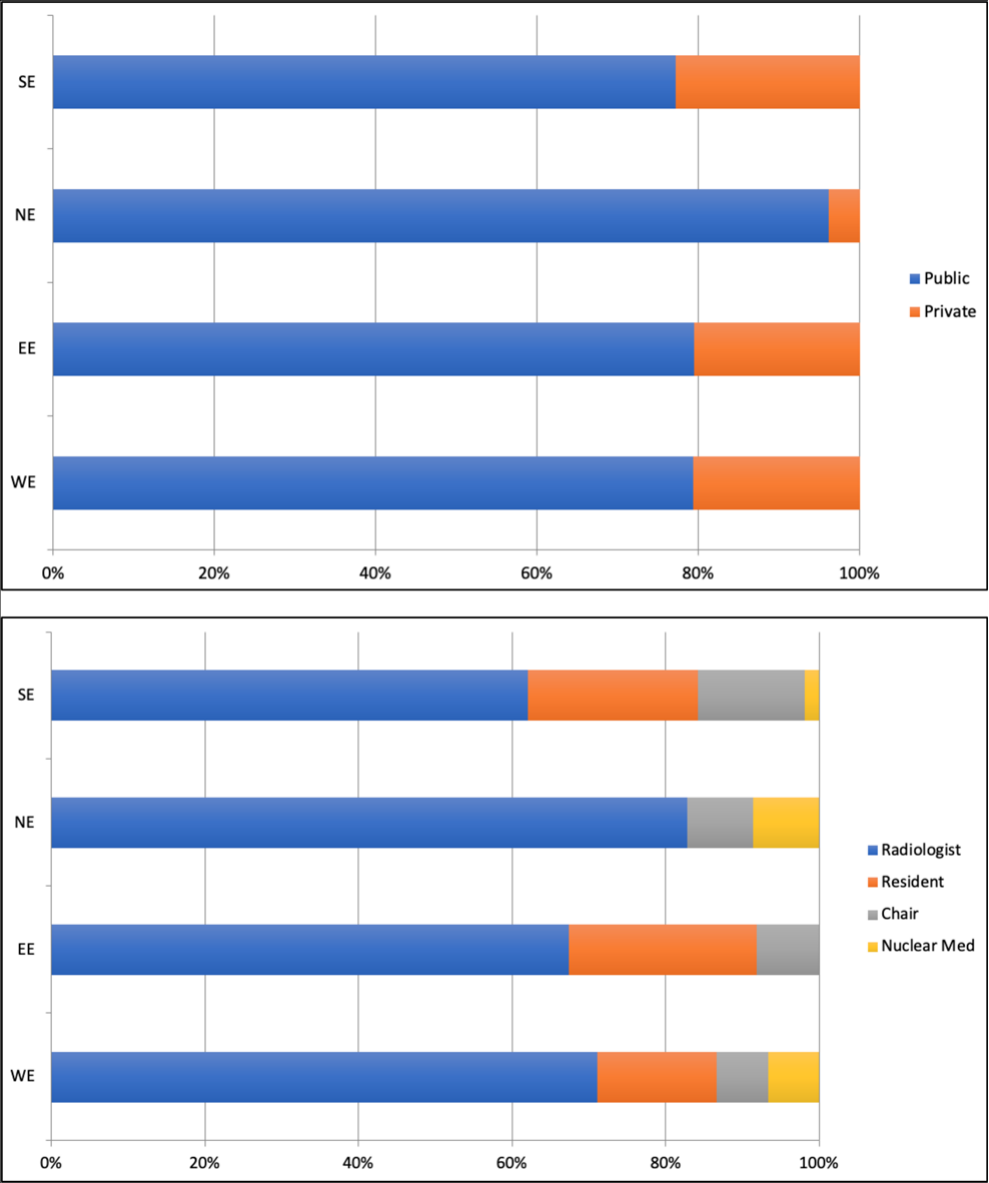
Distribution between public and private (top diagram) and the working role (bottom diagram) of the European radiologists joining the survey. Europe is divided into Eastern Europe (EE), Northern Europe (NE), Southern Europe (SE) and Western Europe (WE) according to the United Nations geoscheme

A total of 292 ESOI members in good standing for the year 2018 took part in the survey (Fig. 1 and Table 2). Overall, 173 out of 292 (59.2%) respondents worked at university hospitals, 68 (23.3%) were employed in private hospitals, and 51 (17.5%) worked at public non-academic hospitals. Of the respondents, 192 out of 292 (65.7%) were staff radiologists, 54 (18.5%) residents and 32 (11%) chairpersons and the remaining 14 (4.8%) had other roles (i.e. 9 nuclear medicine physicians, 3 consultants and 2 surgeons) (question #1). The demographics of European survey participants in terms of public and private distribution and working role are reported in Fig. 2. Most of the respondents (260 out of 292; 89%) declared that radiologists at their department attend MTBs (question #2); among the 32 radiologists who did not attend MTBs, 21 out of 32 (65.6%) do not participate because they are not formally invited by the MTB coordinator, 7 (21.9%) are invited but do not attend because of their busy schedule for imaging and reporting, and 4 (12.5%) are not interested in participating (question #2a). In most cases (190 out of 260; 73.1%), only one radiologist attends the MTB meeting, whereas in 70 cases (26.9%), two radiologists are present (question #3).

**Table 2:** Number of survey respondents per country

Country	# of respondents
Albania	1
Argentina	2
Armenia	2
Australia	2
Austria	6
Bangladesh	1
Belgium	7
Belarus	2
Bosnia and Herzegovina	1
Brazil	4
Bulgaria	3
Chile	1
China	1
Colombia	4
Costa Rica	1
Croatia	4
Czech Republic	3
Denmark	2
Egypt	2
France	5
Georgia	3
Germany	7
Greece	12
Hungary	5
India	13
Iran	4
Ireland	1
Israel	2
Italy	65
Japan	1
Kazakhstan	1
Lesotho	1
Latvia	1
Lithuania	3
Malta	2
Morocco	1
Netherlands	5
Norway	4
Pakistan	1
Peru	1
Poland	11
Portugal	19
Qatar	1
Romania	9
Russian Federation	8
Serbia	1
Slovakia	1
Slovenia	2
Spain	15
South Africa	3
Sweden	4
Switzerland	4
Trinidad and Tobago	1
Turkey	6
Ukraine	1
UK	12
USA	4
Uruguay	1
Venezuela	1
Vietnam	1
All	292

Out of 260 radiologists attending MTBs, 173 (66.5%) receive the list of patients who will be discussed during the MTB meeting, including imaging studies. Conversely, 33 (12.7%) receive only the list of patients who will be discussed during the MTB meetings without imaging studies, and 54 (20.8%) do not get prepared beforehand because they do not receive the list of patients to be discussed (question #4). However, only 5 (1.9%) and 114 (43.9%) of respondents (260) review all or over 70% of exams prior to MTB meetings, respectively. Moreover, 22 (8.5%), 39 (15%) and 57 (21.9%) of respondents review 50–70%, 30–50% and 10–30% of exams, respectively, whereas 6 respondents (2.3%) do not review any exams and 17 of them (6.5%) do not know (question #4a). The main obstacles to reviewing imaging studies prior to the MTB meetings are the lack (78 out of 221; 35.3%) or poor quality (*n* = 40; 18.1%) of imaging studies of outpatients, and also the lack of time due to a busy schedule for imaging and reporting (*n* = 103; 46.6%); 39 survey participants did not respond (question #4b).

The available facilities for reviewing imaging studies during MTB meetings are PACS workstations with monitor (85 out of 260; 32.7%), PACS workstations connected to a video projector (*n* = 143; 55%) or portable personal computer (*n* = 32, 12.3%), respectively (question #4c). Radiologists are included in the final multidisciplinary report in 213 cases out of 260 (81.9%) (question #5). If the radiologist’s opinion differs from the primary imaging report, only in 104 cases out of 260 (40%) a supplementary report is provided during MTB meetings (question #6).

Questions from #7 through #9 concern administrative support. Most radiologists attend MTB meetings during their regular working hours (187/260; 71.9%) (question #7) and their attendance is addressed in the appraisal process of the department (*n* = 174; 66.9%) (question #8). MTB meetings are accredited with continuing medical education (CME) in 39 cases only (15%) (question #9).

**Fig. 3:**
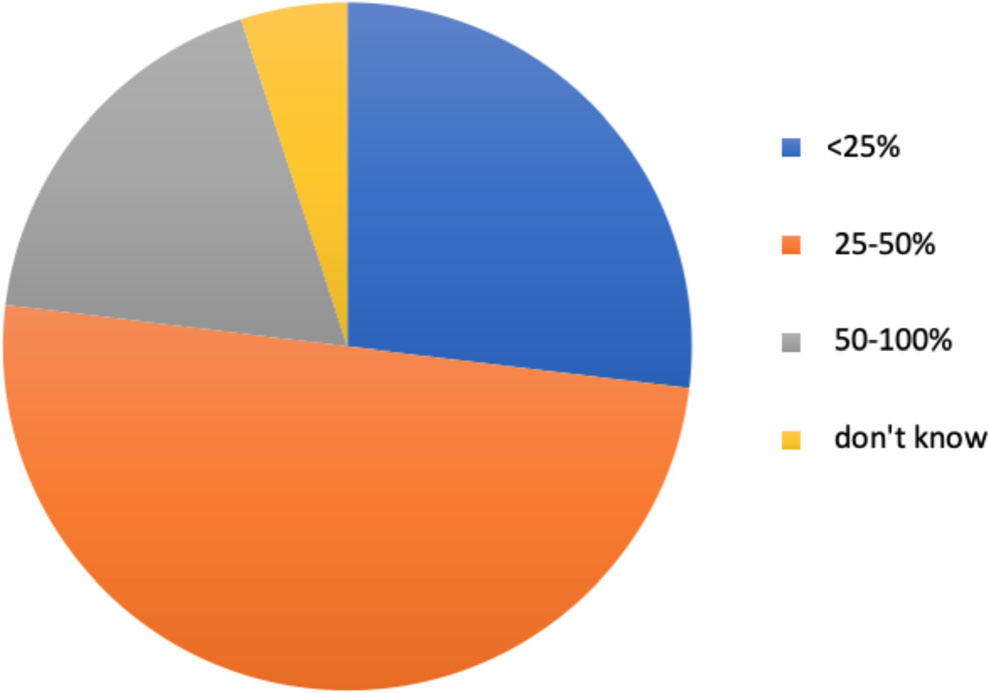
Percentage of cases in which the diagnostic strategy or therapeutic decision has changed due to the participation of radiologists in MTBs, according to the European radiologists joining the survey. 50–100% of cases, 18.1%; 25–50% of cases, 50%; < 25% of cases, 26.9%; do not know, 5%

According to the respondents, the attendance of radiologists at the meetings changes the diagnostic strategy or refine the therapeutic decisions in a range of 25–50% of cases discussed for 130 out of 260 (50%) respondents, and in more than 50% of cases for 47 of them (18.1%), while 70 participants (26.9%) stated that this percentage is less than 25% and the remaining 13 (5%) do not know (question #10) (Fig. 3).

**Fig. 4:**
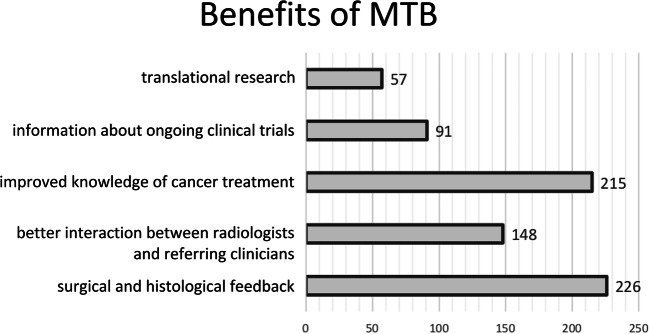
Benefits of MTB meetings as perceived by surveyed radiologists. The *x*-axis shows the number of answers

**Fig. 5:**
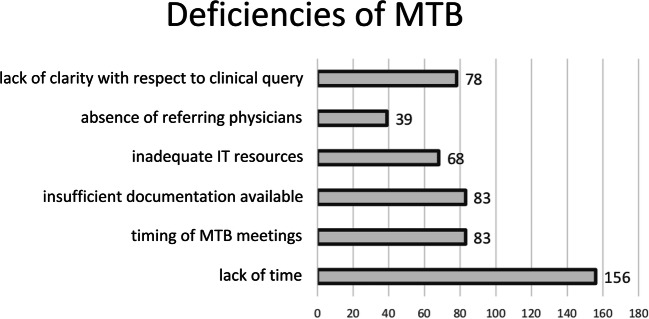
Deficiencies of MTB meetings as perceived by surveyed radiologists. The *x*-axis shows the number of answers

Questions #11 and #12 were aimed at assessing the perceived benefits and deficiencies of MTB meetings for radiologists (Fig. 4 and Fig. 5). According to the survey respondents, MTB benefits include surgical and histological feedback (226/260, 86.9%), better interaction between radiologists and referring clinicians (148/260, 56.9%), improved knowledge of cancer treatment (215/260, 82.7%), information about ongoing clinical trials (91/260, 35%) and translational research (57/260, 21.9%). Conversely, perceived MTB deficiencies were lack of time (156/260, 60%), timing of MTB meetings (83/260, 31.9%), insufficient documentation available (83/260, 31.9%), inadequate IT resources (68/260, 26.1%), lack of clarity with respect to clinical query (78/260, 30%) and absence of referring physicians (39/260, 15%). Of note, 90.4% (235/260) of respondents reported that the patient is not present at MTB meetings, whereas for 10 (3.8%) and 15 (5.8%) respondents, the patient is sometimes present or always present, respectively (question #13). MTBs were held in the morning (74 of respondents, 28.5%), early afternoon (82/260, 31.5%), or late afternoon (35/260, 13.5%), during lunchtime (50/260, 19.2%) or at different time points for the remaining 19 (7.3%) (question #14).

Overall, involvement in MTBs was mandatory for 153 out of 260 (58.8%) of the respondents, and very useful for 107 of them (41.2%). None of the respondents thought that MTBs are not useful (question #15).

## Discussion

To our knowledge, this is the first European survey on the role of the radiologist and the related issues in MTBs.

Most of the respondents worked in a university hospital (59.2%), and only 11% (32/292) of them were not involved in MTBs (of whom 65.6% because not formally invited, and only 12.5% because not interested in participating). This is in line with the fact that currently the radiologist has a fundamental role in the MTB as a “core member”. According to the RCR document, all “core members” must personally attend two out of three MTBs [11]. Moreover, the radiologist needs adequate time to review images before the meeting to provide a robust radiological opinion of the cases and avoid errors. In order to do this, it is necessary to have the list of patients to be discussed during the MTB, at an agreed minimum time in advance, and all the imaging studies performed by the patient (also at other hospitals) should be available.

The time required for a radiologist to review the images of a case reported by him/herself is different from that necessary to review a case reported by another colleague, or even multiple examinations performed in different hospitals. For this reason, examinations performed at other hospitals should be available within an appropriate length of time before the meeting. However, our findings show that only 43.9% of radiologists reviewed over 70% of exams prior to MTB meetings, and imaging studies are reviewed by one-third of radiologists in less than 50% of cases. Possible explanations for this include the fact that only 66.5% of respondents received the list of patients and the imaging studies before the meeting, the lack of time due to a busy schedule for imaging and reporting (46.6%), and the difficulty of reviewing poor-quality imaging studies performed elsewhere (18.1%). In this context, an inaccurate or lacking review of imaging studies before the meeting can lead to significant errors in patient care.

According to the RCR document [11], there should be at least two radiologists designated for each site-specific meeting to provide continuous support, but our findings show that in 73.1% of cases, the MTB meeting is attended by one radiologist only. In general, radiologists deal with different types of cancer and therefore have to attend several meetings in a week, which can make their time commitment especially hard. In addition, many meetings are outside normal working hours or during lunchtime, and in different locations from the normal working place, with consequent problems related to travelling and time management. Problems in MTB attendance have been emphasised in the literature [2], and to this regard it is worth mentioning that an American Society of Clinical Oncology (ASCO) survey showed that although multidisciplinary attendance occurred 70% to 86% of the time, many respondents still did not have access to MTBs and/or lacked certain types of specialists at their institutions, with a small but significant fraction of respondents (24.6%) attending MTBs at nearby institutions [1]. A potential solution might be the implementation of dedicated hardware and/or software platforms to manage MTBs from remote locations, but meeting rooms should be equipped with appropriate technology, and in any case, the issues of lack of time and inconvenient meeting times would remain unresolved [12]. According to our survey, meetings were held mainly in the morning (28.5%) or early afternoon (31.5%), or during lunchtime (19.2%), probably in relation to the needs of the members of the different MTB meetings.

All of the time spent reviewing images, writing supplementary reports and attending meetings should be accounted for as regular working hours and addressed in the appraisal process of the department. However, according to our survey, only in 71.9% of cases the radiologist’s involvement was accounted for in normal working hours and in 66.9% of cases, it was addressed in the appraisal process of the department.

From the radiologist’s point of view, meeting rooms need to have appropriate technology to project high-resolution images (55%), as well as PACS facilities with image reviewing workstations to display imaging studies and eventually retrieve prior examinations (32.7%).

According to the RCR paper [11], the radiologist who has reviewed the images must document that he has done so, independently of whether his/her opinion is in agreement or not with the previous report. This supplementary report, which could influence the clinical decision-making of patients, should be available to MTB members, either at the time of the decision or in the next days before the beginning of treatment. However, our survey shows that only 40% of respondents provide a supplementary report when their opinion differs from the primary imaging report, although in 81.9% of cases radiologists are included in the final multidisciplinary report.

Another important point concerns the presence of patients during meetings. In a survey of over 2000 cancer health professionals in the UK, the majority of them felt that it was neither desirable nor practical to include patients in MTB meetings [13]. However, it is important to ensure that patients are informed about MTBs in a way that allows them to be actively engaged in the decision-making process [14, 15]. Based on our findings, patients did not participate in MTB discussions in 90.4% of cases.

In the respondents’ opinion, the participation of radiologists in MTB meetings is mandatory (58.8%) or very useful (41.2%), and its most important benefits are surgical and histological feedback (86.9%), improved knowledge of cancer treatment (82.7%) and better interaction between radiologists and referring clinicians for discussing rare cases (56.9%). Actually improved communication between health professionals is a recognised putative benefit of MTB working [2], which enables radiologists to assume a more active role in patient care by taking part in team decision-making and allows clarifying the diagnostic strategy or refining therapeutic decisions of clinician members [16]. In the respondents’ view, the attendance of radiologists at the meetings could change the diagnostic strategy or refine the therapeutic decisions in a range of 25–50% of cases discussed (50%). This finding is in line with data from the ASCO survey, revealing that MTB working led to a change of 1% to 25% in treatment plans for 44% to 49% of patients with breast cancer and for 47% to 50% of patients with colorectal cancer. The same survey showed that MTBs were associated with 25% to 50% changes in surgery type and/or treatment plans for 14% to 21% of patients with breast cancer and for 12% to 18% of patients with colorectal cancer [1].

Moreover, involvement in MTB meetings is a good opportunity for trainees to learn, and for members to update their professional knowledge, yet in 85% of cases, they are not CME accredited. Involvement in MTB meetings is essential for both improved patient care and medical research, as well as for continuing education. Members who are actively involved in multidisciplinary discussions have the opportunity to keep themselves updated with ongoing developments of state-of-the-art oncology and clinical studies conducted at their centre. Also, the vast majority (96%) of respondents to the ASCO survey agreed that MTBs can have a teaching value [1]. However, currently, the significant time expenditure required for preparation and performance of MTB sessions finds no counterpart in adequate reimbursement and CME accreditation. Since the demand for MTB sessions will be further increasing, considerable efforts are urgently needed to ensure that the radiology service is adequately acknowledged.

A limitation of our survey is that its specific nature might have led to a selection bias due to collecting data solely from members of a subspecialty radiological society, leaving out opinions from a potentially much larger number of radiologists sharing the same activities and related issues. This might limit the generalisability of our findings and possibly underestimate any shortcomings related to MTB organisation that could occur outside the working institutions of the radiologists involved in our survey. A further potential limitation is the relatively higher prevalence in our survey of respondents from one country (i.e. Italy) compared with other countries, which might introduce a bias in the results towards the Italian system.

In conclusion, our survey (conducted within a selected group of radiologists with a special interest in oncologic imaging) has revealed several criticisms that need to be solved in order to ensure that the presence of a radiologist in MTBs can yield a real added value both to the radiologist and the entire MTB team.

## Abbreviations

ASCO: American Society of Clinical Oncology
CME: Continuing medical education
ESOI: European Society of Oncologic Imaging
MTB: Multidisciplinary tumour board
PACS: Picture archiving and communication system
RCR: Royal College of Radiologists
